# Antiproliferative Activity of Antibiotics through DNA Binding Mechanism: Evaluation and Molecular Docking Studies

**DOI:** 10.3390/ijms24032563

**Published:** 2023-01-29

**Authors:** Alexandros-Dimitrios C. Magklaras, Christina N. Banti, Sotiris K. Hadjikakou

**Affiliations:** Laboratory of Biological Inorganic Chemistry, Department of Chemistry, University of Ioannina, 45110 Ioannina, Greece

**Keywords:** biological inorganic chemistry, antitumor activity, antibiotics, structure activity relationship (SAR), DNA binding studies

## Abstract

The antiproliferative activity of three antibiotics clinically use, was studied through DNA inhibition mechanisms, ex vivo, in silico and in vitro. The ex vivo interaction of DNA with ciprofloxacin hydrochloride (**CIP·HCl**), penicillin G sodium salt (**PEN·Na**), and tetracycline hydrochloride (**TC·HCl**) was determined by UV-Vis spectra and viscosity measurements. Furthermore, their binding constants (*K_b_*) toward CT-DNA were calculated (*K_b_ =* (2.8 ± 0.6) × 10^4^ (**CIP·HCl**), (0.4 ± 0.1) × 10^4^ (**PEN·Na**) and (6.9 ± 0.3) × 10^4^ (**TC·HCl**) Μ^−1^). Docking studies on the binding interactions of antibiotics with DNA were performed to rationalize the ex vivo results. The in vitro antiproliferative activity of the antibiotics was evaluated against human breast adenocarcinoma (MCF-7) cells (IC_50_ values: 417.4 ± 28.2 (**CIP·HCl**), >2000 (**PEN·Na**) and 443.1 ± 17.2 (**TC·HCl**) μΜ). Cell cycle arrest studies confirmed the apoptotic type of MCF-7 cells. The toxicity of the studied agents was in vitro tested against human fetal lung fibroblast cells (MRC-5). The results are compared with the corresponding one for doxorubicin (**DOX**). Despite their low binding affinity to DNA (*K_b_*) or their different mode of interaction, **TC·HCl** (anthracycline) or **CIP·HCl** (quinolones), exhibit notable antiproliferative activity and low toxicity.

## 1. Introduction

Worldwide, cancer remains a major health problem and cause of death, despite the scientific progress that has been achieved [1,2]. Based on modern cell biology, cancer is progressing through the mechanisms of cancer stem cell replication, the uncontrolled growth and migration of cells with a dysregulated cell cycle and their ability to continuously self-renew [2]. After the COVID-19 era, a rise in cancer incidence rates is expected [1]. Therefore, this fact will induce the increase in cancer mortality rates [1]. Thus, there is a great need for the development of new efficient chemotherapeutics with low toxicity.

Antibiotics are another class of chemotherapeutic compounds [3]. Antibiotics are isolated from secondary metabolites produced either by a microorganism, such as bacteria, or by a higher organism, such as a higher plant [3]. However, they are primarily derived from the bacteria *Streptomyces* [3]. These metabolites exhibit anti-pathogenic properties, inhibiting the proliferation of other living cells [2]. Mostly, antibiotics are used to treat infections, but they have also made a significant contribution to cancer treatment since the 1960s [4]. It is thought that, compared to other anti-cancer agents, cytotoxic antibiotics, such as doxorubicin (**DOX**), show greater selectivity against cancer cells than healthy ones [4]. Their effects against cancer cells come from three main mechanisms, which are anti-proliferative, anti-epithelial-mesenchymal-transition and pro-apoptotic [2]. Antibiotics can eliminate cancer cells by killing them at any stage of the cell cycle, even in the G_0_ phase [2]. They can also promote programmed cell death, apoptosis, by targeting the cancer suppressor gene p53, caspase-3/8/9, the apoptotic genes B cell lymphoma-2 (Bcl-2) and pro-Bcl-2-associated X (Bax) [2]. Moreover, they can contribute against metastasis by inhibiting cancer cell metastasis, acting as regulators of epithelial-mesenchymal-transition [2]. Antitumor antibiotics can act either by intercalating with DNA strongly or by damaging it [2,3,4].

Nowadays, among the most used antibiotics are quinolones, beta-lactams, and tetracyclines [2]. Ciprofloxacin is a member of the second-generation fluoroquinolone family of antibiotics [2,5,6,7]. It exhibits bacteriostatic activity and shows high efficacy against both Gram-negative and -positive pathogenic bacteria [5]. In mammalian cells, its mechanism of action has been associated with the inhibition of the enzyme topoisomerase II [2,8,9]. Its inhibition is achieved when a high concentration of antibiotic is administered, but even with a lower concentration cell proliferation it is interrupted [8]. This has led researchers to attribute the cytotoxicity of the antibiotic to other mechanisms of action, such as through mitochondrion and/or cell membrane [8]. Ciprofloxacin can cause alterations in mitochondrial DNA by inhibiting its synthesis following a reaction with the topoisomerase II isoform in the mitochondrion and depleting ATP stores [8]. Thus, apoptosis is induced to the cell through an intracellular pathway [8]. Penicillins belong to the beta-lactam class of antibiotics [10,11,12,13] and make up almost half of today’s antimicrobial agents [11]. The most widely used penicillin is benzyl-penicillin (penicillin G) [14]. The structure of penicillin consists of a linked β-lactam ring with a thiazolidine ring and a side chain [12]. More specifically, in the case of penicillin G, a benzyl molecule constitutes the side chain [14]. Its potent antimicrobial activity, lies primarily in the chemical reactivity of the four membered β-lactam ring, due to its inner tension [11]. Penicillin G shows activity only against Gram-positive bacteria and not against Gram-negative ones [10]. Its mechanism of action has not been fully elucidated, but it inhibits the synthesis of the bacterial cell wall [10,12,14] and activates the endogenous autolytic system [10,12]. Tetracycline is a member of the tetracycline family of antibiotics [15], which is used successfully against infections caused by either anaerobic or aerobic Gram-positive and -negative bacteria [16,17,18]. Structurally, tetracycline is isomorphic to doxorubicin showing extensive conformational relationship. Tetracycline binds covalently the 30S subunit of the ribosome, preventing the binding of aminoacyl t-RNA to the ribosome [18,19,20]. In this way, the process of protein synthesis in prokaryotes is inhibited [15,19]. The mechanism of protein synthesis in prokaryotes and that of eukaryotic mitochondria show similarities [15]. This has led to the view that tetracycline may interfere with protein synthesis taking place in the mitochondria of mammalian cells [15].

**Scheme 1:**
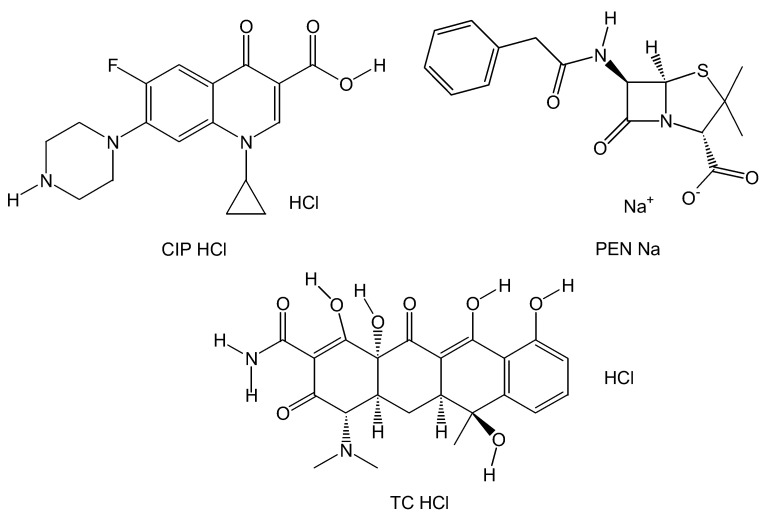
Molecular structures of **CIP·HCl**, **PEN·Na**, and **TC·HCl**.

During our study toward the development of new chemotherapeutics against breast cancer, we evaluated the interaction of (i) ciprofloxacin hydrochloride (**CIP·HCl**) (Scheme 1, (ii) penicillin G sodium salt (**PEN·Na**) (Scheme 1), and (iii) tetracycline hydrochloride (**TC·HCl**) (Scheme 1) with DNA. DNA binding affinity to the antibiotics was examined by ultraviolet-visible (UV-Vis) spectroscopy and viscosity measurements. The binding constants (*K_b_*) of the antibiotics toward Calf Thymus-DNA (CT-DNA) were calculated. CT-DNA is a model DNA with many base pairs and no sequence specificity [21]. In addition, docking studies were performed in order to rationalize the experimental results. **DOX** was used as a control drug for comparison. Finally, **CIP·HCl**, **PEN·Na** and **TC·HCl** were evaluated for their in vitro antiproliferative activity against human breast adenocarcinoma (MCF-7) cell line. **DOX** is a drug in clinical use for the treatment of human breast cancer, and this is why **CIP·HCl**, **PEN·Na** and **TC·HCl** were evaluated against MCF-7 cells. The relative toxicity among the antibiotics was evaluated against human fetal lung fibroblast cells (MRC-5).

## 2. Results and Discussion

### 2.1. General Aspects

Stock solutions (10^−2^ M) of the commercial forms of the antibiotics **CIP·HCl**, **PEN·Na**, and **TC·HCl** in double distilled water (ddH_2_O) were used for the study of the DNA-drug interaction and for the cell proliferation tests. The results are compared with those of **DOX**.

### 2.2. Ex Vivo Studies

#### DNA Binding Studies

DNA remains the main target of any chemotherapeutic drug [4]. Agents such as antibiotics bind to DNA through either covalent bond with nitrogen atom of a base of DNA, such as guanine N7 [22] and/or through non-covalent interactions, such as electrostatic, intercalative, or groove binding of the agent with the DNA helix and along the minor or major groove [22]. The interaction of the antibiotics **CIP·HCl**, **PEN·Na**, and **TC·HCl** toward CT-DNA was investigated by UV-Vis absorption spectroscopy and viscosity measurements.

*UV-Vis absorption spectroscopy*: The binding properties of DNA inhibitors were studied by absorption spectroscopy [22]. The hypochromism or hyperchromism observed in the UV spectra of CT-DNA-binder and free CT-DNA is associated with the conformation of the DNA double-helix structure [22]. Thus, hyperchromism is assigned either to the cleavage of the hydrogen bonds, which stabilized the secondary structure of DNA, or to groove binding [22], while the hypochromism is attributed to the intercalative or electrostatic binding mode [22]. It is well known that one of the most immediate indicators of possible intercalative binding to DNA is hypochromicity associated with electron transitions of an intercalating molecule [22]. Specifically, the following changes occur on intercalation: unwinding and elongation of the DNA helix, electronic interaction of the intercalator within the helix, and stiffening and orientation of the intercalator within the DNA helix [22].

**Figure 1:**
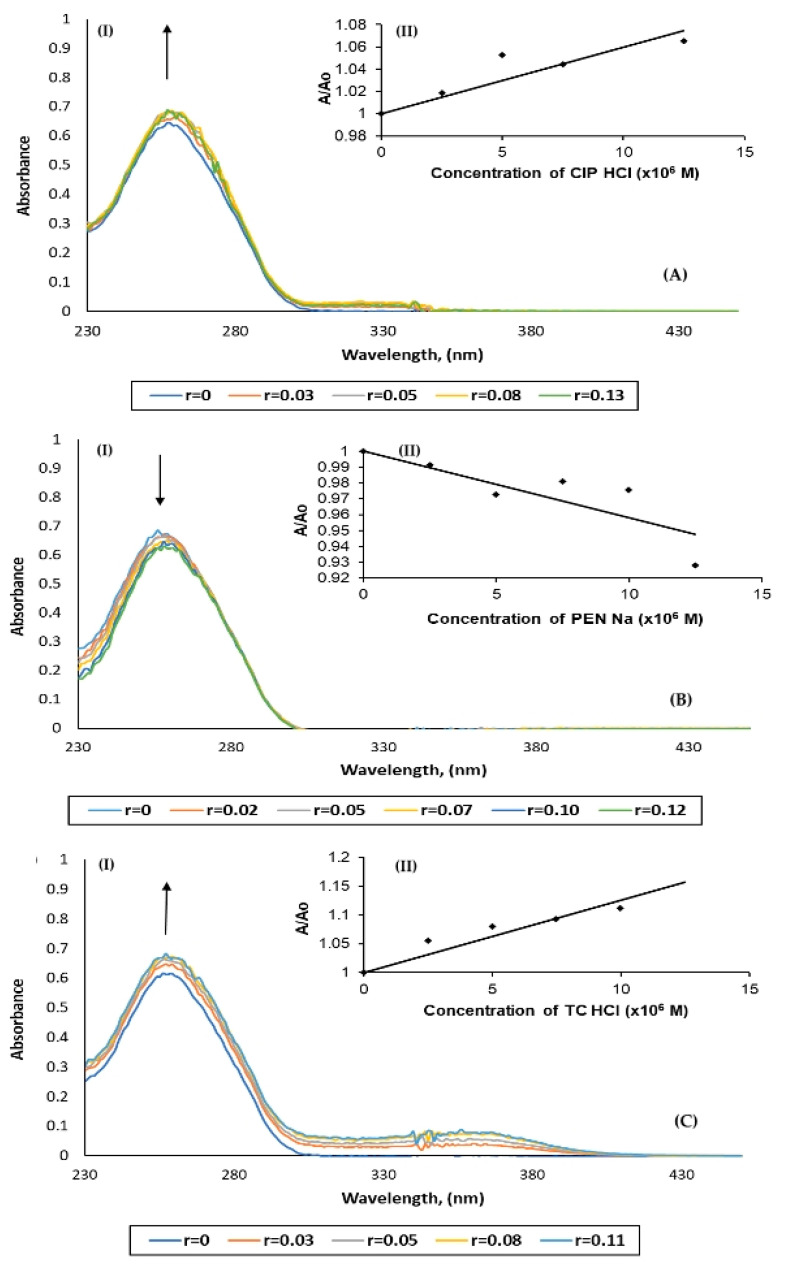
(I) UV spectra of CT-DNA in buffer solution in the absence and presence of (**A**) **CIP·HCl**, (**B**) **PEN·Na**, and (**C**) **TC·HCl** at r values (r = [compound]/[DNA], r = 0, 0.03, 0.05, 0.08, 0.13, and [DNA] = 9.7 × 10^−5^ M (**CIP·HCl**), r = 0, 0.02, 0.05, 0.07, 0.10, 0.12, and [DNA] = 10.0 × 10^−5^ M (**PEN·Na**) and r = 0, 0.03, 0.05, 0.08, 0.11, and [DNA] = 9.4 × 10^−5^ M (**TC·HCl**)) and (II) plot of A/A_o_ vs. [compound] at λ_max_ = 257 nm (A/A_o_ = DNA solution absorbance in the presence of DNA-binder (A) and without DNA-binder (A_o_)).

Figure 1 shows the UV spectra of buffer solution of CT-DNA, at constant concentration, in the absence and presence of **CIP·HCl**, **PEN·Na**, and **TC·HCl** at various r values (r = [compound]/[DNA], [DNA] = 9.7 × 10^−5^ M (in the case of **CIP·HCl**), 10.0 × 10^−5^ M (in the case of **PEN·Na**) and 9.4 × 10^−5^ M (in the case of **TC·HCl**)), while the A/A_o_ versus [compound] at λ_max_ = 257 nm plots are also shown. In the cases of the **CIP·HCl** and **TC·HCl**, a hyperchromism was observed by 3.3% and 8.5%, respectively (Table 1). Therefore, the groove binding mode of the **CIP·HCl** and **TC·HCl** was concluded. On the contrary, a hypochromism was observed in the case of **PEN·Na** by 1.4%, suggesting its intercalative mode of interaction with DNA.

**Figure 2:**
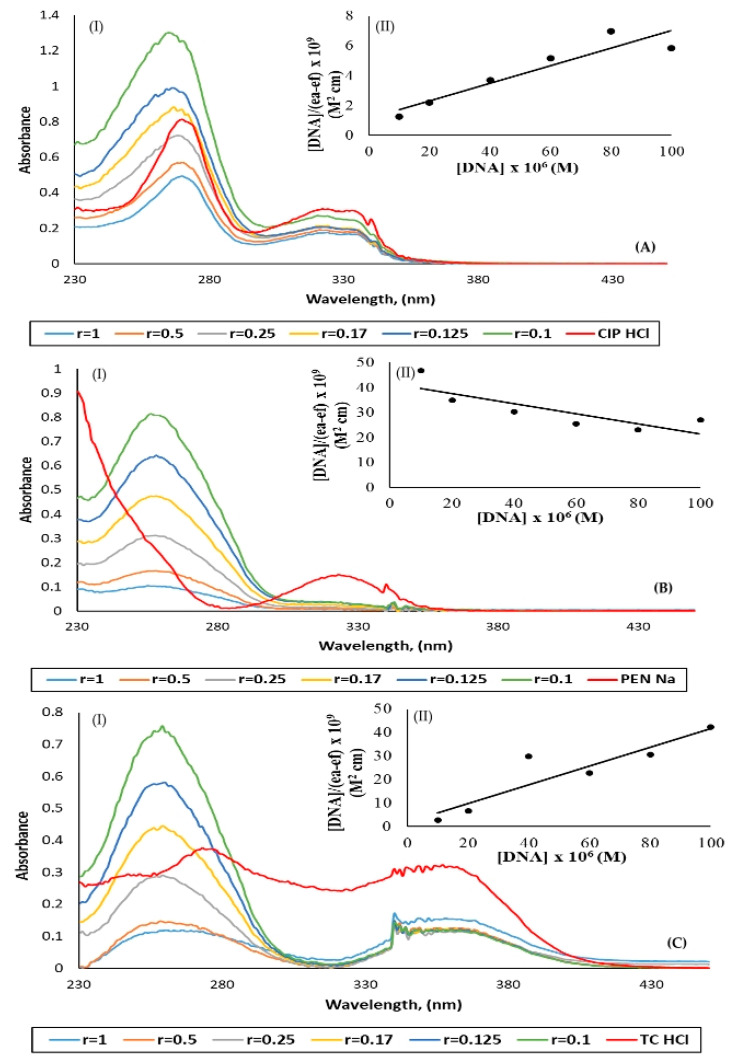
(I) UV spectra of complexes **CIP·HCl** (**A**), **PEN·Na** (**B**), and **TC·HCl** (**C**) in the absence and presence of CT-DNA at r values of 1, 0.5, 0.25, 0.17, 0.125, and 0.1 (r = [compound]/[DNA], [compound] = 10 μM, [CT-DNA] = 10–100 μM). (II) Graphical plot of [DNA]/(e_a_ − e_f_) vs. [DNA].

The binding constant (*K_b_*) of the antibiotics to CT-DNA was determined by evaluating the changes in the UV-Vis absorbance spectra of (a) [**CIP·HCl**] = 10^−5^ M at 310–320 nm, (b) [**PEN·Na**] = 10^−5^ M at 310–320 nm, and (c) [**TC·HCl**] = 10^−5^ M at 370–380 nm with increasing concentrations of CT-DNA. Table 1 summarizes the DNA binding constants (*K_b_*) for **CIP·HCl, PEN·Na**, and **TC·HCl**. *K_b_* is given by the ratio of the slope to the y-intercept in the graphs [DNA]/(ε_A_−ε_f_) vs. [DNA] (Figure 2) according to the Equation (1) [23]. The binding constant (*K_b_*) of the antibiotics to CT-DNA was checked at 310–320 nm because the absorbance of free DNA solutions is negligible and therefore any absorbance that appears is due to the DNA-binder complex formation. The minor variations in the DNA-binder solution absorbances are due to their weak interaction. Especially when **PEN·Na** is used, almost no interaction occurred, which results in to the insignificant value of Kb.

(1) $$\frac{\left[DNA\right]}{\left(\varepsilon_{A}-\varepsilon_{f}\right)}=\frac{\left[DNA\right]}{\left(\varepsilon_{b}-\varepsilon_{f}\right)}+\frac{1}{K_{b}(\varepsilon_{b}-\varepsilon_{f})}$$

where [DNA] stands for the concentration of CT-DNA, ε_A_ equals A_obsd_/[compound], ε_f_ is the extinction coefficient for the free compound, and ε_b_ is defined as the extinction coefficient for the compound in fully bound form.

The highest binding constants were observed for **TC·HCl** (6.9 ± 0.3) × 10^4^ M^−1^. It is followed by the corresponding constant for **CIP·HCl**, which was calculated as (2.8 ± 0.6) × 10^4^ M^−1^. Finally, **PEN·Na** shows the lowest *K_b_* equal to (0.4 ± 0.1) × 10^4^ Μ^−1^. The order of *K_b_* values is: **TC·HCl** > **CIP·HCl** > **PEN·Na.** Despite the presence of π, π* orbitals located in the aromatic ring of **PEN·Na**, which may interact with the DNA bases, its binding constant is smaller than that of the classical intercalators and metallointercalators, where the binding constant was reported to be in the order of 10^7^ M^−1^ [24]. This is due to the low depth of penetration of the intercalator between the base pairs of DNA [25]. Moreover, the reported *K_b_* values for the typical classical intercalator ethidium bromide (EB), which is the positive control of the measurements, is 4.94 × 10^5^ M^−1^ (in 25 mM Tris-HCl/40 mM NaCl buffer, pH 7.9) [26].

*Viscosity measurements*: The DNA solution viscosity measurement method determines the interaction of a drug with the DNA molecule as a function of the hydrodynamic changes caused by the binding agent [27]. When DNA is incubated with the antibiotic, its length is altered, and this affects the viscosity of its solution. When an agent intercalates in the DNA strands, it causes an increase in both its length and viscosity. No significant difference in viscosity is observed in the case of electrostatic interaction with DNA. Finally, a decrease in both the length and viscosity of DNA is observed when an agent cleaves its strands or when it covalently binds to DNA causing bending of the helix. Hence, it is shown that viscosity exhibits high sensitivity to changes in DNA and is widely used to determine how a compound binds to DNA [27,28,29]. The correlation between the relative viscosity of the DNA solutions (*n/n*_0_) and the length of DNA (*L/L*_0_) is given by Equation (2) [27,28].

*L*/*L_0_* = (*n*/*n*_0_)^1/3^ (2)

**Figure 3:**
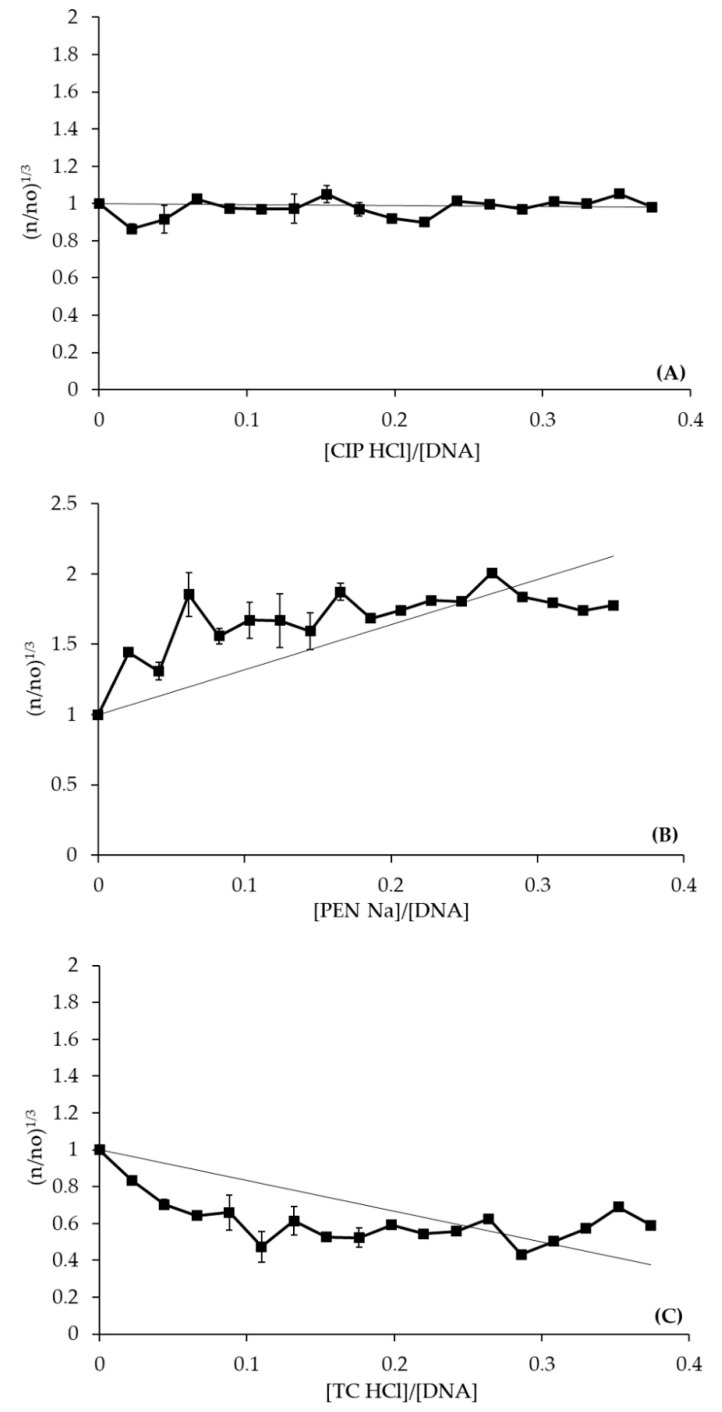
Relative viscosity of CT-DNA with increasing concentrations of (**A**) **CIP·HCl** (r = 0.37), (**B**) **PEN·Na** (r = 0.35), (**C**) **TC·HCl** (r = 0.37) ([DNA] = 10 mM, r = [compound]/[DNA], *n* is the viscosity of DNA in the presence of the compounds and *n_o_* is the viscosity of DNA alone).

The CT-DNA solution, at a concentration of 10 mM, is incubated with the increasing amounts of antibiotics so that the molecular ratio [compound]/[DNA] reaches r = 0.37 in cases of **CIP·HCl**, **TC·HCl**, and r = 0.35 in case of **PEN·Na**. Figure 3 shows the relative specific viscosity (*n/n*_0_)^1/3^ versus the [compound]/[DNA] ratio. The trend of lowering in the DNA solution viscosity with the increasing concentrations of the antibiotics **CIP·HCl** and **TC·HCl** suggests either groove binding or DNA cleavage. In contrast, an increasing in the DNA solution viscosity observed after successively increasing concentrations of the **PEN-Na** suggests intercalation between DNA strands. 

### 2.3. In Silico Studies

#### Computational Studies—Molecular Docking

In an attempt to rationalize the factors that govern the DNA–antibiotic interaction, molecular docking computations were employed. AutoDock 4.2 software was used to detect the binding energies of the tested **CIP·HCl**, **PEN·Na**, and **TC·HCl** to the DNA (PDB: www.rcsb.org/structure/1bna (accessed on 1 November 2022). 1BNA is a dodecamer with a sequence of d(CGCGAATTCGCG)_2_. 1BNA (Protein Data Bank database), which is used in this work, is a dodecameric structure (d(CGCGAATTCGCG)2) of a complete right-handed B-DNA turn, and it is used because of the absent of the structure of CT-DNA in the PDB. The CT-DNA, on the other hand, simulates DNA with many base pairs and no sequence specificity. With the aim to perform the experiments as close as possible to those occurring in the real biological systems, the CT-DNA was used for the experimental studies. Due to the lack of CT-DNA structure, however, in the PDB, the docking studies were performed with the above-mentioned dodecamer. Based on the ex vivo studies for DNA-agent interaction, a groove binding mode was concluded for **CIP·HCl** and **TC·HCl**, while **PEN·Na** intercalates to DNA. The optimized binding energies calculated for the experimental docking poses of **CIP·HCl**, **TC·HCl**, and **PEN·Na** toward DNA are −5.41, −6.11, and −6.13 kcal/mol, while the corresponding value for **DOX** is −9.36 kcal/mol (**CIP·HCl ≈ TC·HCl** < **PEN·Na** < **DOX**). The K_b_ values follow the order: **PEN·Na** < **CIP·HCl** < **TC·HCl**. The observed high binding energy in respect to its low binding constant of **PEN·Na** is due to the low depth of penetration in the base pairs of DNA [25].

**Figure 4:**
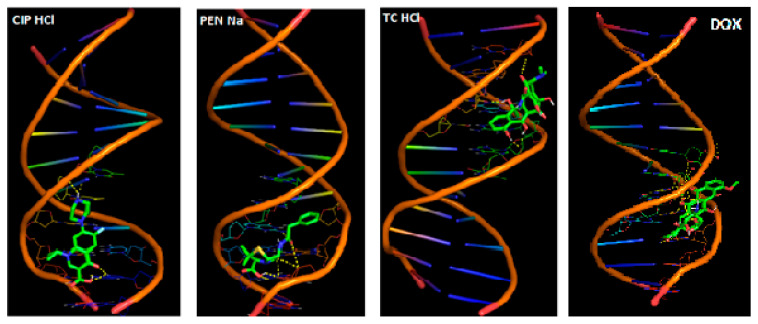
Binding sites of **CIP·HCl**, **PEN·Na**, **TC·HCl** and **DOX** toward DNA (PDB: 1BNA).

Figure 4 shows the docked-out conformations with the best scores analyzed for the interaction type. Table 2 summarizes the details for the nature of the interaction between DNA and drugs.

### 2.4. In Vitro Studies

#### 2.4.1. Anti-Proliferative Activity

The antibiotics **CIP·HCl**, **PEN·Na**, and **TC·HCl** were tested for their in vitro anti-proliferative activity against the hormone-dependent human breast adenocarcinoma cell line MCF-7. Their antiproliferative activity (IC_50_) was calculated after incubation of cancer cells with the drugs for 48 h by the Sulforhodamine B (SRB) assay. This assay relies on the ability of SRB to bind at the amino acid residues of the protein component of the living cells [30]. Table 3 summarizes the IC_50_ values of the antibiotics against MCF-7 cells. The corresponding IC_50_ values of **CIP·HCl** and **TC·HCl** toward MCF-7 cells are 417.4 ± 28.2 and 443.4 ± 17.2 μΜ, respectively. In contrast, no activity was determined for **PEN·Na** within the concentrations tested. **CIP·HCl** shows the strongest activity, followed by **TC·HCl** (Table 3). The IC_50_ values order is **DOX** < **CIP·HCl ≈ TC·HCl <** … **< PEN·Na.** The drugs **CIP·HCl** and **TC·HCl** studied are less active than **DOX** (by 5 fold) (Table 3) [31]. The toxicity of the agents was tested against MRC-5 cells (Table 3). The IC_50_ values of **CIP·HCl, PEN·Na**, and **TC·HCl** toward MRC-5 cells (210 ± 4.4, >2000 and 382.1 ± 31.0 μΜ respectively) reveals lower toxicity than DOX by 208-fold (**CIP·HCl**) and 378-fold (**TC·HCl)** than DOX (Table 3).

#### 2.4.2. Cell Cycle Arrest Study

**Figure 5:**
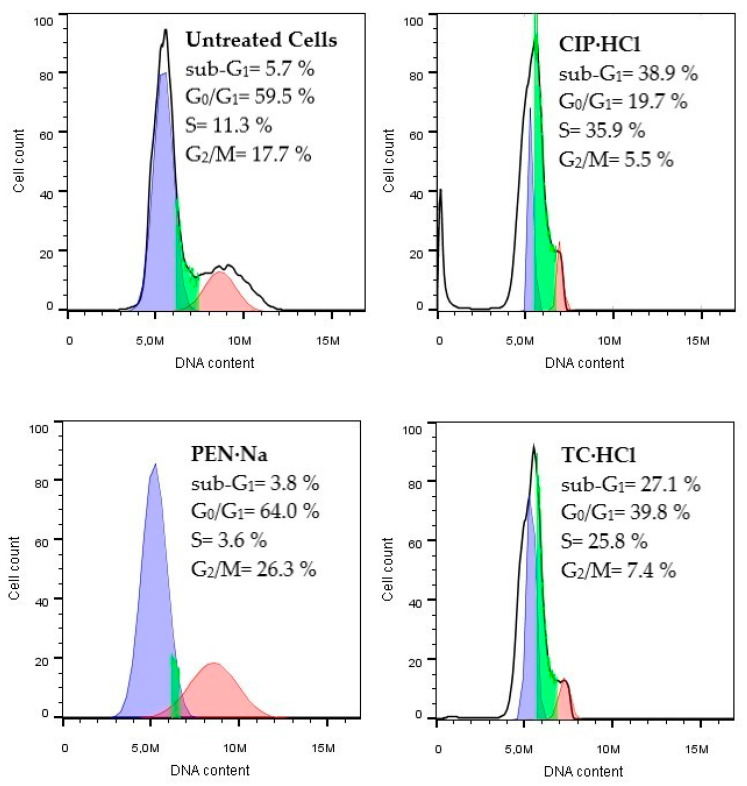
Effect of **CIP·HCl**, **PEN·Na**, and **TC·HCl** on MCF-7 cell cycle. The relative number of cells within each cell cycle was determined by flow cytometry. Number of cells in sub-G_1_, G_0_/G_1_, S, and G_2_/M phases are indicated.

Cell cycle arrest studies were employed in order to confirm the apoptotic type of MCF-7 cells upon their tretment with **CIP·HCl**, **PEN·Na**, and **TC·HCl**, which requires either direct or indirect interaction of DNA with the binders. MCF-7 cells were treated for 48 h with **CIP·HCl**, **PEN·Na**, and **TC·HCl** at their IC_50_ values, stained with propidium iodide (PI), and the amount of DNA was analyzed by flow cytometry. The results are presented in the DNA frequency histograms as the number of cells versus DNA content in different phases of cell cycle (sub-G_1_, G_0_/G_1_, S, and G_2_/M) (Figure 5).

The untreated MCF-7 cells population in the sub-G_1_, G_0_/G_1_, S, and G_2_/M phases is 5.7%, 59.5%, 11.3%, and 17.7%, respectively. However, in the case of treated MCF-7 cells with **CIP·HCl** and **TC·HCl**, the distribution in the sub-G_1_ phase is increased at 38.9 and 27.1 %, respectively, indicating an increase in the number of apoptotic cells [32,33]. However, in the case of **PEN·Na**, the percentage of the sub-G_1_ phase remains unchanged in respect to the untreated cells. This is expected since the **PEN·Na** shows no inhibition activity on the proliferation of the MCF-7 cells (IC_50_ > 2000 μΜ). Moreover, the percentage of the treated MCF-7 cells with **CIP·HCl** and **TC·HCl** is increased at 35.9 and 25.8 % at the S phase, respectively, in contrast to the untreated cells (11.3%), suggesting the suppression of the cell proliferation due to DNA synthesis inhibition caused by **CIP·HCl** and **TC·HCl** [32]. On the other hand, **PEN·Na** causes cell cycle arrest in the G_0_/G_1_ and G_2_/M phases. 

## 3. Conclusions

Doxorubicin (DOX) is a well-known antibiotic with anticancer activity used against various types of cancer [4]. DOX inhibits the protein synthesis by targeting the enzyme topoisomerase II, while it intercalates to DNA [4]. In an attempt to elucidate the mechanism of antiproliferative activity of antibiotics, three drugs were chosen here to be studied for their activity. One acts through topoisomerase II inhibition (**CIP·HCl**), one intercalates to DNA (**PEN·Na**), and one is isomorphic of DOX (**TC·HCl**) [4,13,34].

Binding studies (ex vivo and in silico) show that **CIP·HCl** binds to DNA through the major groove. The groove binding type was also concluded for the DNA interaction with **TC·HCl**, which, however, contradicts with the intercalation mode that has been proposed previously [17,19]. Nevertheless, our findings for **TC·HCl** agree with those proposed by Smythies JR, et al. [20]. **PEN·Na,** on the other hand, intercalates to DNA as **DOX** also does. The *K_b_* values, however, suggest the weak interaction of DNA with **CIP·HCl**, **PEN·Na**, and **TC·HCl**, the strongest one being with **TC·HCl**.

Based on the experimental data on the DNAbinder interaction, the proper poses from those proposed by the docking calculations were chosen. **PEN·Na** and **TC·HCl** dock to DNA with a binding energy value of −6.1 Kcal/mol, while **CIP·HCl** docks with higher binding energy value (−5.4 Kcal/mol). **DOX**, on the other hand, shows the lowest binding energy value to DNA (−9.4 Kcal/mol). Therefore, among the antibiotics studied, **TC·HCl** and **PEN·Na** dock stronger with DNA.

**TC·HCl** and **CIP·HCl** show antiproliferative activity against MCF-7 cells (IC_50_ values 417.4 μΜ and 443.1 μΜ, respectively), while **PEN·Na** exhibits negligible activity that is in accordance with the lowest binding constant to DNA. **DOX** inhibits MCF-7 cells with IC_50_ value 81.0 μM [31]. By comparing the toxicity against MRC-5 cells, **TC·HCl** and **CIP·HCl** are 378-fold and 208-fold less toxic than **DOX** (Table 3) [32]. 

In conclusion, among the antibiotics studied, the structural isomorphic to DOX, **TC·HCl**, shows the stronger binding affinity to DNA, the highest antiproliferative activity, and the lowest toxicity. Thus, neither the mechanism of action nor the type of DNA binding seems to govern the anticancer effectiveness of these antibiotics but the geometric features.

## 4. Materials and Methods

### 4.1. Chemicals and Instruments

All solvents used were of reagent grade, while **TC·HCl** was purchased from Sigma Aldrich. **CIP·HCl** and **PEN·Na** were kindly provided from HELP Pharmaceuticals and COOPER C.A., which are acknowledged. A UV-1600 PC series spectro-photometer of VWR was used to obtain electronic absorption spectra. An Ostwald-type viscometer was used for viscosity measurements.

### 4.2. DNA Binding Studies

#### 4.2.1. Ultraviolet−Visible Studies

This study was carried out as described previously [23]. DNA stock solution was prepared by diluting CT-DNA with buffer (containing 15 mM trisodium citrate and 150 mM NaCl at pH = 7) followed by gentle stirring for 3 days and kept at 4 °C for no longer than a week. The stock solution of CT-DNA gave a ratio of UV absorbance at 260 and 280 nm (A_260_/A_280_) higher than 1.8, indicating that the DNA was sufficiently free of protein contamination. The DNA concentration was determined by the UV absorbance at 260 nm after 1:20 dilution using ε = 6600 M^−1^ cm^−1^. The DNA solutions were used afterwards without any dilution. Given that the DNA solutions were freshly prepared for each experiment, the initial concentration of DNA solution varied between the experiment. For the experiments, UV spectra of CT-DNA in buffer solution in the absence and presence of **CIP·HCl, PEN·Na**, and **TC·HCl** at r values (r = [compound]/[DNA], [DNA] = 9.7 × 10^−5^ M and r = 0, 0.03, 0.05, 0.08, 0.13 (in the case of CIP·HCl addition), 10.0 × 10^−5^ M and 0, 0.02, 0.05, 0.07, 0.10, 0.12 (in the case of PEN·Na addition) and 9.4 × 10^−5^ M and 0, 0.03, 0.05, 0.08, 0.11 (in the case of TC·HCl addition), respectively) were recorded. For binding constant *K_b_* values determination, UV spectra of CIP·HCl, PEN·Na, and TC·HCl in the presence and absence of CT-DNA at r values 1, 0.5, 0.25, 0.17, 0.125, and 0.1 (CIP·HCl, PEN·Na and TC·HCl) (r = [compound]/[DNA], [compound] = 10 μM, [CT-DNA] = 10–100 μM) were also recorded.

#### 4.2.2. Viscosity Measurements

This study was carried out as previously reported [27]. The kinematic viscosity of DNA solutions with or without **CIP·HCl**, **PEN·Na**, and **TC·HCl** (0–0.37 [**CIP·HCl**]/[DNA], 0–0.35 [**PEN·Na**]/[DNA], 0–0.37 [**TC·HCl**]/[DNA] molar ratios) was measured with an Ostwald-type viscometer [27].

### 4.3. Molecular Docking

AutoDock Version 4.2.6 program from the Scripps Research Institute was used for the molecular docking studies [35]. The 3D crystal structure of a d(CGCGAATTCGCG)_2_ dodecamer, B-DNA, was obtained from the RCSB Protein Data Bank [PDB: 1BNA]. In order to avoid hindrance, all the water molecules were deleted, for the search of best binding site results. All non-polar hydrogen atoms were merged, and atomic charges (Gasteiger charges) were added to the DNA molecule by MGL Tools-1.5.7. The structures of **CIP·HCl, PEN·Na**, and **TC·HCl** were downloaded from PubChem (https://pubchem.ncbi.nlm.nih.gov (accessed on 1 November 2022) in three-dimensional SDF format, which was converted into mol2 format by Mercury. After that, the compounds were saved into pdbqt format by using MGL Tools-1.5.7. The rest of the molecular docking parameters were kept as default. The final results were performed by using PyMoL [33].

### 4.4. Sulforhodamine B (SRB) Assay

This study was performed according to the procedure reported previously [30,36]. Cells were plated (100 μL per well) in 96-well flat-bottomed microplates, each containing a number of MCF-7 and MRC-5 cells equal to 6000 and 2000 cells/well, respectively. Cells were incubated for 24 h at 37 °C and they were exposed to **CIP·HCl**, **PEN·Na**, and **TC·HCl** for 48 h (50–2000 μM). Addition of an equal volume (100 μL) of either complete culture medium (control wells) or twice the final concentrations of the agent which have been diluted in the complete culture medium (test wells) was followed. Evaluation of the cytotoxicity of the agents was achieved by means of SRB colorimetric analysis, which provides the absorption rate of surviving cells compared to the absorption rate of control cells (untreated cells). The cell density in each well was kept in 6000 cells/well since the results were obtained in regard to the corresponding of the control experiments (untreated cells) and therefore, by keeping the cell density constant, the effect of the number of cells was eliminated. The constant cell density, on the other hand, at 6000 and 2000 cells/well led to an absorbance to the solutions <1.5, avoiding the limitations of Beer–Lambert law (linear curve of absorbance vs concentration for absorbances up to 1.5–2). Moreover, it is known that minor variations in assay parameters (e.g., medium composition, seeding density, drug storage) do not drastically affect the results [37]. 

### 4.5. Cell Cycle Arrest

This study was performed as previously reported [32]. MCF-7 cells were seeded at a density of 1 × 10^5^ cells/well in six-well plates at 37 °C for 24 h. The cells were treated with the antibiotics at their IC_50_ values, and they were incubated for 48 h. The cells were trypsinized, washed twice with PBS, and separated by centrifugation afterwards. The cells were incubated overnight at −20 °C following the addition of 1 mL of cold 70% ethanol. For analysis, the cells were centrifuged, transferred into PBS, incubated with Rnase (0.2 mg/mL) and propidium iodide (0.05 mg/mL) for 40 min at 310 K, and then analyzed by flow cytometry. For each sample, 10,000 events were recorded. The resulting DNA histograms were drawn and quantified using the FlowJo software (version FlowJo X 10.0.7r2).

## Figures and Tables

**Table 1:** The DNA binding constants (*K_b_*) for **CIP·HCl**, **PEN·Na**, and **TC·HCl**.

Antibiotic	*K_b_* (M^−1^)	Hyper-/Hypochromic Effect	H (%)
**CIP·HCl**	(2.8 ± 0.6) × 10^4^	Hyperchromism	3.3
**PEN·Na**	(0.4 ± 0.1) × 10^4^	Hypochromism	1.4
**TC·HCl**	(6.9 ± 0.3) × 10^4^	Hyperchromism	8.5

**Table 2:** Calculated bonds between interacting atoms of the DNA nucleotides (d(CGCGAATTCGCG)_2_) and the antibiotics.

Antibiotic	Binding Energy (kcal/mol)	DNA Strand	Nucleotide	Nucleotide’s Atom	Atoms in Compound	Distance (Å)
**CIP·HCl**	−5.41	B	dG14	H7	O2	2.242
		A	dT8	O2	H41	1.892
**PEN·Na**	−6.13	A	dG10	H21	O2	1.669
		B	dG16	H22	O5	2.205
**TC·HCl**	−6.11	A	dA6	O3	H51	2.038
		B	dC21	O2	H42	2.009
		A	dA6	O3	H56	1.809
**DOX**	−9.36	A	dG10	H3	O7	1.872

**Table 3:** IC_50_ values of the antibiotics and doxorubicin against adenocarcinoma breast cell line MCF-7 (hormone-dependent) and normal human lung fibroblast cell line MRC-5.

Compounds	IC_50_ Values (μΜ)		TPI	Ref.
	MCF-7	MRC-5		
**CIP·HCl**	417.4 ± 28.2	210 ± 4.4	0.5	*
**PEN·Na**	>2000	>2000	-	*
**TC·HCl**	443.1 ± 17.2	382.1 ± 31.0	0.9	*
**DOX**	80.95	1.01	0.01	[31,32]

* Present work; TPI = therapeutic potency index: IC_50_(MRC-5)/IC_50_(MCF-7).

## Data Availability

All data are availability from authors upon request.
